# Prognostic stratification of patients with coronary artery stenosis by presence or absence of left anterior descending artery lesions

**DOI:** 10.1007/s12928-026-01259-1

**Published:** 2026-03-03

**Authors:** Eiji Shibahashi, Kensuke Shimazaki, Yusuke Inagaki, Takehiro Hata, Shintaro Haruki, Hisao Otsuki, Junichi Yamaguchi, Kentaro Jujo

**Affiliations:** 1 Department of Cardiology, Tokyo Women’s Medical University, Tokyo, Japan; 2 Department of Cardiology, Saitama Medical Center, Saitama Medical University, Saitama, Japan

**Keywords:** Percutaneous coronary intervention; Left anterior descending coronary artery; Long-term prognosis

## Abstract

Patients with coronary artery disease (CAD) typically develop atherosclerosis proximal to the left anterior descending coronary artery (LAD). However, it is unclear how CAD location affects long-term prognosis in the era of drug-eluting stents. This study compared the clinical profiles and long-term outcomes of patients with and without LAD lesions after percutaneous coronary intervention (PCI). Of 2,497 consecutive patients with CAD who underwent PCI, 1,245 who underwent first-time PCI were enrolled. Baseline profiles and long-term prognoses, including major adverse cardiovascular events (MACE), were compared between patients with de novo CAD localized to the LAD (n=986) and non-LAD lesions (n=259). MACE included cardiovascular death, nonfatal myocardial infarction, and target vessel revascularization. Patients in the LAD group were more likely to be older; had an increased prevalence of diabetes, dyslipidemia, and impaired cardiac and renal function; and had significantly more multi-vessel disease, calcified lesions, and long lesions than the non-LAD group. Kaplan-Meier analysis revealed that the MACE rate was significantly higher in the LAD group (16.6% vs. 5.4%, p<0.001) regardless of the presence of the left main disease. LAD lesions were associated with an increased rate of MACE independent of other prognostic factors (hazard ratio: 2.26, 95% confidence interval: 1.19–4.28). Patients with LAD lesions had worse atherosclerotic profiles at baseline; this was associated with a poorer prognosis after the initial PCI. These findings indicate the importance of early detection and management of LAD lesions in improving the long-term prognosis of patients with CAD.

**Graphical Abstract:**

Prognostic comparisons of in-hospital mortality and MACE after PCI in patients with and without LAD lesions. Comparison of in-hospital mortality and MACE between patients with and without LAD lesions. LAD, left anterior descending artery; MACE, major adverse cardiovascular event; PCI, percutaneous coronary intervention.

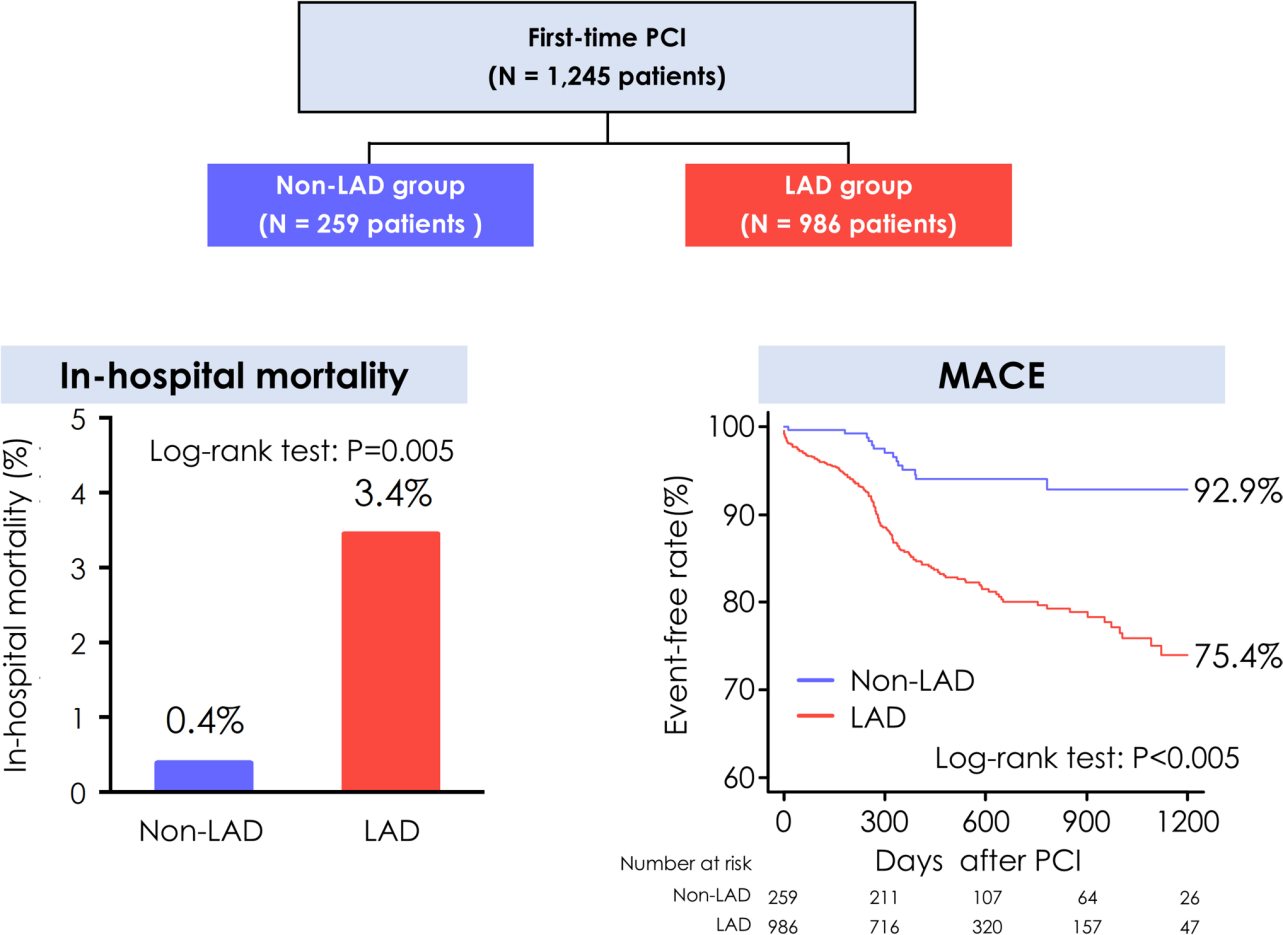

**Supplementary Information:**

The online version contains supplementary material available at 10.1007/s12928-026-01259-1.

## Introduction

Coronary atherosclerosis generally begins with proximal lesions in the left anterior descending artery (LAD), partly because of major branching sites with turbulent flow patterns [1, 2]. The LAD usually supplies blood flow to a large portion of the myocardium. Therefore, acute myocardial infarction (MI) with lesions in either the left main coronary artery (LMCA) or proximal LAD is associated with increased risks of major adverse cardiovascular (CV) events (MACE) and mortality [3–5]. However, atherosclerotic stenosis isolated to non-LAD coronary arteries is frequently observed during routine coronary angiography. The prognoses and clinical profiles based on the initial location of coronary artery atherosclerosis have not been fully compared. Although non-LAD lesions are relatively rare localized lesions, it remains unclear whether LAD lesions are truly associated with a poor prognosis.

Previous studies have reported comparable long-term prognoses after percutaneous coronary intervention (PCI) in patients with proximal and non-proximal LAD lesions [6–8]. According to recent improvements in the long-term PCI prognosis, the 2018 European Society of Cardiology/European Association for Cardio-Thoracic Surgery guidelines recommend PCI or coronary artery bypass grafting (CABG) for single-vessel disease in the LAD as class I [9, 10]. However, despite insufficient evidence comparing long-term outcomes after PCI for patients with LAD lesions with those for patients with non-LAD lesions, PCI is recommended for non-LAD lesions unless a multivessel disease is present [10]. Previous studies comparing whether the presence or absence of LAD lesions affects the long-term prognosis of PCI have not excluded patients who underwent prior PCI (i.e., patients with non-LAD lesions may include those with prior PCI for LAD lesions). This can easily lead to misinterpretation of the relationship between the presence or absence of LAD lesions and prognosis. Based on this background, the current study aimed to compare the baseline profiles and clinical outcomes of patients with LAD and non-LAD lesions by selecting patients undergoing PCI for the first time.

## Methods

### Study population and endpoints

**Fig. 1:**
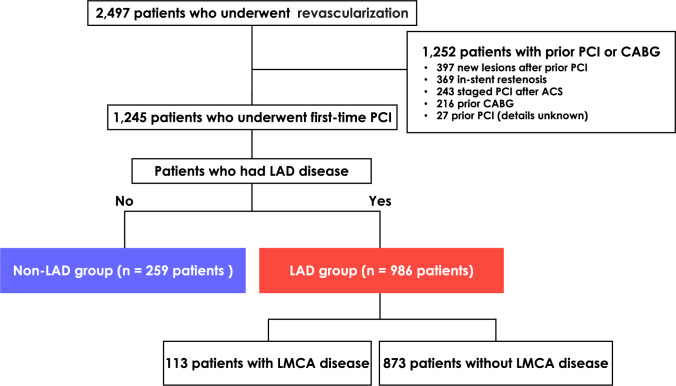
Patient flow chart. CABG, coronary artery bypass grafting; LAD, left anterior descending artery; LMCA, left main coronary artery; PCI, percutaneous coronary intervention

This observational cohort study included 2,497 consecutive patients who underwent PCI at Tokyo Women’s Medical University between January 2010 and August 2015. After excluding 1,252 patients who had undergone prior PCI/CABG, 1,245 patients undergoing PCI for the first time were finally enrolled in this study. Patients with a history of myocardial infarction that had been managed medically (without prior PCI or CABG) were not excluded. The study was registered in the University Hospital Medical Information Network Clinical Trials Registry under the identifier UMIN000048607. The enrolled patients were divided into two groups based on the presence of significant angiographic stenosis or occlusion in the LAD lesion: the LAD (986 patients) and non-LAD (259 patients) groups (Fig. 1). In this study, all significant LAD lesions in the LAD group were subsequently treated with PCI**.** Patients with LMCA lesions were assigned to the LAD group. Each PCI-treated vessel was counted separately. Patients with LAD lesions were included in the LAD group irrespective of whether PCI for the LAD lesion was performed during the primary procedure or as a staged PCI. The primary endpoint of this study was the incidence of MACE. The secondary clinical endpoints were death from any cause, CV death, nonfatal MI, target vessel revascularization (TVR), and in-hospital mortality after index PCI. To evaluate the prognostic impact of LMCA stenosis, patients with LAD lesions were further divided into two subgroups: (i) those with LMCA and (ii) those without LMCA. The prognostic impact of different clinical presentations of acute coronary syndrome (ACS) and chronic coronary syndromes (CCS) in each subgroup was examined. In addition, the duration and type of dual antiplatelet therapy were left to the discretion of the cardiologist. The study protocol was in accordance with the regulations of the hospital ethics committee and was approved by the institutional review board (Approval No. 3503-R). Patient enrollment was performed in accordance with the principles of the Declaration of Helsinki.

### Definitions

MACE included CV death, nonfatal MI, and TVR. Nonfatal MI was defined based on the fourth universal definition of MI types 1, 2, and 4b [11]. CV deaths were classified according to established criteria [12], which included deaths attributed to acute MI, sudden cardiac death, heart failure, stroke, CV procedures, CV hemorrhage, or other specified CV causes. TVR was defined as any repeat revascularization in the target vessel, including PCI and CABG in the target vessel. Significant stenosis was defined as ≥ 70% diameter narrowing by visual estimation in a major epicardial artery, ≥ 50% in the LMCA, or total occlusion [13]. Multivessel disease was defined as ≥ 70% stenosis in at least two major coronary arteries, excluding LMCA [14]. LMCA lesions were categorized within the LAD group. Coronary calcification was defined as readily apparent radiopacities within the vascular wall at the stenosis site, visible before contrast injection [15]. TVR was performed if angiographic stenosis > 75% on the target lesion by eyeballing assessment, in addition to cardiac ischemia-related symptoms and/or positive results on a cardiac stress test, fractional flow reserve test ≤ 0.80, treadmill exercise testing, or myocardial perfusion scintigraphy. The final decision to perform TVR was left to the attending physicians. The authors used a per-patient basis rather than a per-lesion basis for the clinical events. Coronary artery disease (CAD) was classified as ACS, including ST-segment elevation MI (STEMI), non-STEMI, unstable angina pectoris, and CCS, including effort angina pectoris and silent myocardial ischemia.

### Data collection and follow-up

Patients’ baseline characteristics were evaluated, including (i) age; (ii) sex; (iii) body mass index; (iv) history of coronary risk factors (hypertension, diabetes, dyslipidemia, and smoking habits), heart failure, peripheral artery disease, atrial fibrillation, stroke, or MI; (v) laboratory data (hemoglobin, albumin, total bilirubin, blood urea nitrogen, serum creatinine, estimated glomerular filtration rate (eGFR), sodium, C-reactive protein, brain natriuretic peptide, lipid profiles, and hemoglobin A1c); (vi) clinical presentation of CAD; and (vii) oral medication at the time of PCI. The left ventricular ejection fraction (LVEF) was measured using transthoracic echocardiography or contrast ventriculography during the index hospitalization. These parameters were compared between the LAD and non-LAD groups. After PCI, outpatient visits were scheduled every 2 months. The follow-up period was defined as the time from the index PCI to the last confirmed clinical contact or event (e.g., outpatient visit, telephone follow-up, or medical record review). The duration of follow-up was determined based on the longest period during which clinical outcome data could be reliably collected under the protocol approved by the institutional ethics committee. The endpoints were compared between the groups during the observation period.

### Interventional procedures

PCI was performed according to the standard procedures in contemporary practice. The balloon catheter or stent size, inflation pressure, and use of intravascular imaging modalities, including intravascular ultrasound and optical coherence tomography, were left to each operator’s discretion. Successful stent implantation was defined as < 20% residual stenosis angiographically assessed by eyeballing at the end of the procedure [16]. The endpoint of the PCI procedure was left to each operator’s discretion under the guidance of angiography and/or imaging modalities. Without any contraindications, patients received dual antiplatelet therapy (aspirin and an oral P2Y12 inhibitor) for ≥ 6 months, followed by lifelong single antiplatelet therapy. Some patients underwent angiographic follow-up 9–12 months after index PCI.

### Statistical analyses

Continuous variables were summarized as median [interquartile range (IQR)], and categorical variables were expressed as numbers and percentages. Between-group comparisons of continuous variables were performed using the Wilcoxon rank-sum test (Mann–Whitney U test), and categorical variables were compared using the chi-square test or Fisher’s exact test, as appropriate. A p-value < 0.05 was considered statistically significant. Primary and secondary endpoints were evaluated using Kaplan–Meier curves, and differences among the groups were assessed using the log-rank test or log-rank trend test. Event rates for long-term outcomes were calculated using the Kaplan–Meier method, and values were presented as estimated cumulative incidences at 2 years. Univariate Cox hazard regression analyses were performed to evaluate the association between stenosis in the LAD lesions and prognosis after PCI. Variables were considered clinically significant if they had a p-value of < 0.1 in the univariate model, and such variables were included in the multivariable model. Multivariable Cox hazard regression analysis was performed to exclude confounding factors and identify independent risk factors for the primary endpoint, such as comorbidity of diabetes, history of heart failure, low LVEF (< 40%), administration of angiotensin-converting enzyme inhibitors or statins, presence of LAD lesions, multivessel disease, coronary calcification, and long lesion length (> 20 mm). To further assess LAD involvement's impact on outcomes, we conducted a sensitivity analysis stratifying patients into three groups: Non-LAD (no significant LAD stenosis), LAD (Only) (isolated LAD stenosis), and LAD (Multivessel) (LAD stenosis plus at least one other major coronary vessel). Statistical significance was set at p-values < 0.05. Statistical analyses were performed using the R software, version 3.3.0 (R Foundation for Statistical Computing, Vienna, Austria).

## Results

The patient flowchart is shown in Fig. 1. Ultimately, 1,245 patients who underwent PCI for the first time were analyzed. Of these, 986 (79.2%) had LAD lesions, and 259 (20.8%) did not. The median follow-up period was 514 days (IQR: 330–789). Angiographic follow-up was performed in 511 patients (41.0% of the total population) 9–12 months after the index PCI.

### Baseline profiles

The clinical profiles of the patients are shown in Table 1. The average age of the study population was 67 years, and 75% of the patients were males. Regarding CV risk factors, the prevalence of dyslipidemia, diabetes, and chronic kidney disease was significantly higher in the LAD group than in the non-LAD group. Mean LVEF was significantly lower in the LAD group. Regarding the clinical presentation of CAD, the distributions of ACS, STEMI, and prior MI were comparable between the two groups. Differences in laboratory data were observed for eGFR and HbA1c levels. HbA1c was analyzed separately in diabetic and non-diabetic patients, showing a significant difference only among diabetic patients.

**Table 1:** Baseline profiles

	LAD group	Non-LAD group	P value
	n = 986	n = 259	
Age, years	68 [61–76]	66 [58–74]	< 0.01
Age > 75 years, (%)	287 (29.1%)	61 (23.6)	0.09
Male, (%)	740 (75.1)	189 (73.0)	0.47
Body mass index, kg/m^2^	23.8[21.5–26.2]	23.5 [21.3–26.4]	0.82
Smoking, (%)	553 (56.1)	157 (60.6)	0.20
Hypertension, (%)	751 (76.5)	204 (78.8)	0.46
Dyslipidemia, (%)	661 (67.3)	157 (60.6)	0.047
Diabetes mellitus, (%)	556 (56.6)	116 (44.8)	< 0.01
IDDM, (%)	200 (20.4)	39 (15.1)	0.06
Atrial fibrillation, (%)	90 (9.1)	20 (7.7)	0.54
CKD grade 3–5, (%)	362 (36.7)	72 (27.8)	< 0.01
Hemodialysis, (%)	145 (14.7)	42 (16.2)	0.56
Prior myocardial infarction, (%)	127 (13.0)	35 (13.5)	0.84
Prior heart failure, (%)	155 (15.8)	35 (13.5)	0.39
Clinical presentations			
ACS, (%)	387 (39.2)	89 (34.3)	0.15
STEMI, (%)	139 (14.1)	41 (15.8)	0.99
LVEF, %	51 [43–58]	54 [47–60]	< 0.01
LVEF < 40%, (%)	111 (11.3)	18 (6.9)	0.051
Laboratory Data			
Serum creatinine, (mg/dL)	0.84 [0.73–1.07]	0.82 [0.71–0.98]	0.09
eGFR	63.3 [49.8–77.4]	69.1 [55.0–79.2]	0.01
Total cholesterol, mg/dL	177 [151–206]	168 [150–198]	0.12
LDL cholesterol, mg/dL	104 [81–124]	98 [80–116]	0.24
HDL cholesterol, mg/dL	51 [42–62]	50 [41–61]	0.37
Triglycerides, mg/dL	129 [87–187]	127 [87–193]	0.80
HbA1c (DM patients), %	6.9 [6.3–7.7]	7.2 [6.6–8.1]	0.04
HbA1c (non-DM patients), %	5.8 [5.5–6.0]	5.7 [5.4–6.0]	0.58
CRP, mg/dL	0.16 [0.06–0.42]	0.19 [0.07–0.63]	0.40
BNP, pg/ml	65.9 [27.4–215.5]	73.8 [30.7–154.3]	0.91
Prehospital medications			
ACE-i, (%)	47 (4.8)	11 (4.3)	0.87
ARB, (%)	429 (43.5)	123 (47.5)	0.26
β blocker, (%)	337 (34.3)	78 (30.1)	0.24
Calcium channel blockers, (%)	403 (40.9)	122 (47.1)	0.08
Diuretics, (%)	152 (15.4)	39 (15.1)	0.92
Statin, (%)	402 (40.8)	100 (38.6)	0.57
Warfarin, (%)	75 (7.6)	20 (7.7)	0.99
DOAC, (%)	7 (0.7)	1 (0.4)	0.99
Rate of CAG follow-up, (%)	405 (41.1)	106 (40.9)	0.99

ACE-i = angiotensin converting enzyme inhibitor; ACS = acute coronary syndrome; ARB = angiotensin receptor blocker; BNP = brain natriuretic peptide; CAG = coronary angiography; CKD = chronic kidney disease; CRP = C-reactive protein; DM = diabetes mellitus, DOAC = direct oral anticoagulants; eGFR = estimated glomerular filtration rate; HbA1C = hemoglobin A1C; HDL = high density lipoprotein; IDDM = insulin dependent diabetes mellitus; LAD = left anterior descending coronary artery; LDL = low density lipoprotein; LVEF = left ventricular ejection fraction; STEMI = ST-elevation myocardial infarction

The angiographic and procedural parameters during PCI are shown in Table 2. In the LAD group, 100%, 6.3%, 19.6%, and 22.8% of the patients underwent PCI for the LAD, LMCA, left circumflex artery (LCx), and right coronary artery (RCA), respectively, including both primary and staged procedures. In the non-LAD group, 41.3% of the patients underwent PCI for the LCx and 73.0% for the RCA. Patients in the LAD group had significantly higher rates of multivessel disease, calcified lesions, and long lesions than those in the non-LAD group. Compared with the non-LAD group, the LAD group had a significantly smaller median stent diameter (3.0 [IQR: 2.5–3.5] vs. 3.0 [2.75–3.5] mm, p < 0.001), a longer median stent length (28 [18–35] vs. 23 [16–32] mm, p < 0.001), and a higher distribution of the total number of stents per patient (median 1 [1–2] vs. 1 [1–2], p < 0.001). There was no significant difference in the success rate of the first PCI between the two groups.

**Table 2:** Procedural characteristics

	LAD group	Non-LAD group	P value
	n = 986	n = 259	
PCI lesion			< 0.01
Left Main coronary artery, (%)	62 (6.3)	0 (0)	
Left anterior descending artery, (%)	986 (100)	0 (0)	
Left Circumflex artery, (%)	193 (19.6)	107 (41.3)	
Right Coronary artery, (%)	225 (22.8)	189 (73.0)	
Treatment with DES	781 (79.2)	196 (75.7%)	0.23
Multivessel disease, (%)	613 (62.2)	61 (23.6)	< 0.01
Calcification, (%)	199 (20.2)	33 (12.7)	< 0.01
CTO, (%)	65 (6.6)	25 (9.7)	0.11
Long lesion (> 20 mm), (%)	469 (47.6)	86 (33.2)	< 0.01
Small vessel (< 3.0 mm), (%)	466 (47.3)	107 (41.3)	0.09
Stent diameter, mm	**3.0 [2.5–3.5]**	**3.0 [2.75–3.5]**	** < 0.01**
Stent length, mm	**28 [18–35]**	**23 [16–32]**	** < 0.01**
Number of stents	**1.0 [1.0–2.0]**	**1.0 [1.0–2.0]**	** < 0.01**
IVUS, (%)	436 (69.6)	109 (64.5)	0.23
OCT, (%)	56 (9.0)	15 (8.8)	> 0.99
Rotational atherectomy, (%)	46 (4.7%)	6 (2.3%)	0.12
Procedural success of PCI, (%)	924 (93.7)	247 (95.4)	0.38

CTO = chronic total occlusion; DES = drug eluting stent; IABP = intra-aortic balloon pumping; IVUS = intravascular ultrasound; LAD = left anterior descending coronary artery; OCT = optical coherence tomography; PCI = percutaneous coronary intervention; STEMI = ST-elevation myocardial infarction

### In-hospital and long-term prognosis

**Fig. 2:**
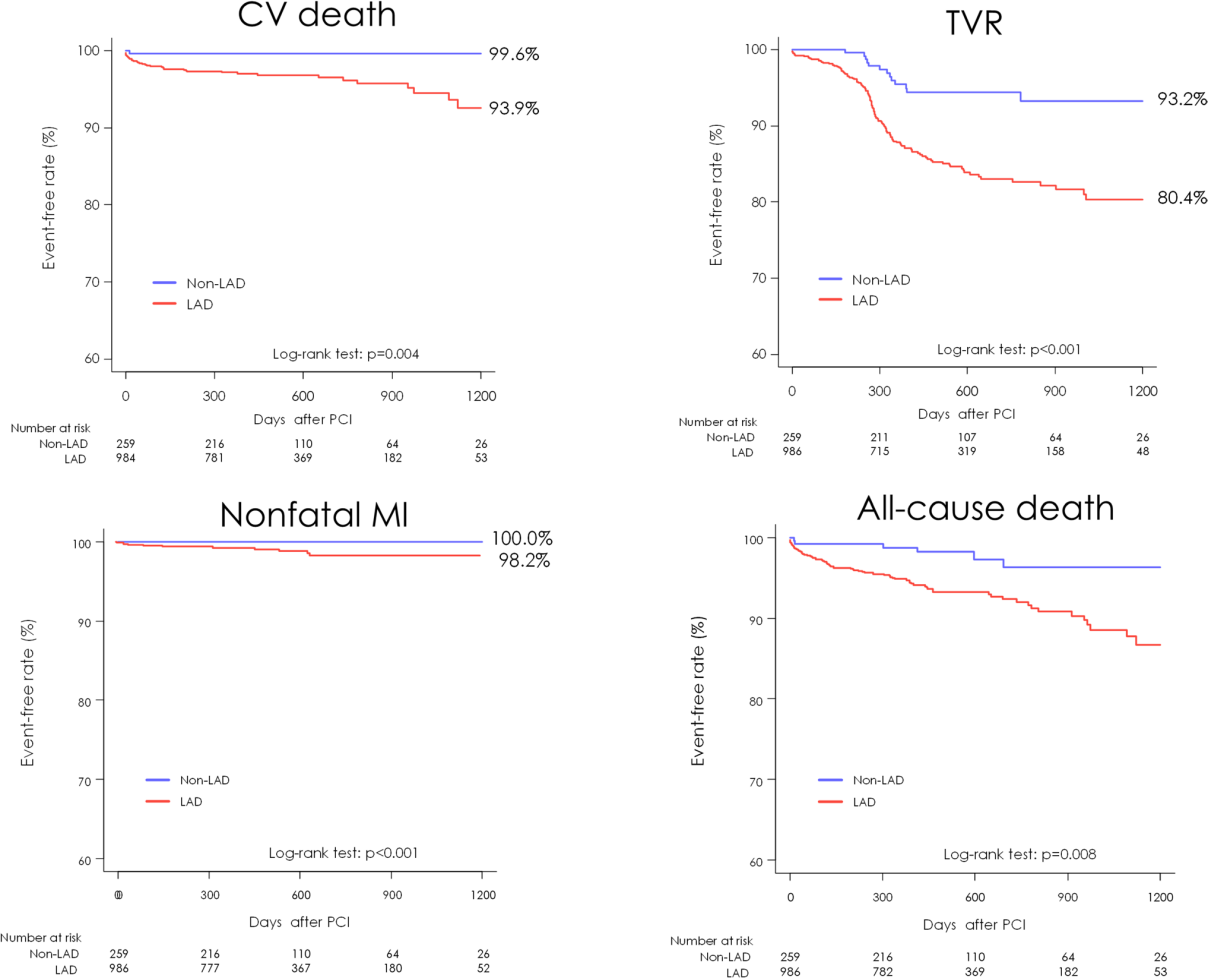
Prognostic comparisons after PCI between patients with and without LAD lesions. Comparison of MACE, CV death, TVR, nonfatal MI, and all-cause death between patients with and without LAD lesions. CV, cardiovascular; LAD, left anterior descending artery; MACE, major adverse cardiovascular event; MI, myocardial infarction; PCI, percutaneous coronary intervention; TVR, target vessel revascularization

The in-hospital mortality was significantly higher in the LAD group than in the non-LAD group (3.4% vs. 0.4%; p = 0.005) (Graphical Abstract). Kaplan–Meier analysis demonstrated significantly higher 2-year estimated rates of MACE, CV death, TVR, and all-cause death in the LAD group than in the non-LAD group (MACE: 19.9% vs. 6.0%, p < 0.001; CV death: 3.5% vs. 0.4%, p = 0.004; TVR: 17.0% vs. 5.6%, p < 0.001; all-cause death: 7.6% vs. 3.7%, p = 0.006 (Graphical Abstract, Fig. 2, and Table 3).

**Table 3:** Clinical outcomes according to the presence of LAD lesions (Kaplan–Meier 2-year estimates)

	LAD group	Non-LAD group	P value
	n = 986	n = 259	
MACE*, (%)	19.9% (16.9–22.9%)	6.0% (2.8–9.2%)	< 0.001
Cardiovascular death, (%)	3.5% (2.2–4.8%)	0.4% (0.0–1.2%)	0.004
Nonfatal MI, (%)	1.9% (0.7–3.0%)	0.0% (0.0–0.0%)	0.067
TVR, (%)	17.0% (14.1–19.8%)	5.6% (2.5–8.7%)	< 0.001
All-cause death, (%)	7.6% (5.6–9.6%)	3.7% (0.5–6.7%)	0.006
In-hospital death, (%)	34 (3.4)	1 (0.4)	0.005

LAD = left anterior descending coronary artery; MACE = major adverse cardiac events; MI = myocardial infarction; TVR = target vessel revascularization

^*^ Major adverse cardiac events consist of cardiovascular death, nonfatal myocardial infarction and target vessel revascularization

Furthermore, the multivariable analysis confirmed LAD lesions as independent predictors of MACE (hazard ratio (HR): 2.42, 95% confidence interval (CI): 1.28–4.58), along with comorbid diabetes (HR: 1.62, 95% CI: 1.10–2.38), low LVEF (HR: 1.80, 95% CI: 1.18–2.73), and coronary calcification (HR: 2.26, 95% CI: 1.56–3.27; Table 4).

**Table 4:** Cox regression model for the major adverse cardiovascular events

Variables	Univariate analysis		Multivariable analysis	
	HR (95% CI)	P value	HR (95% CI)	P value
Age	1.00 (0.99–1.01)	0.95		
Male	1.44 (0.99–2.09)	0.06		
Body mass index	0.99 (0.95–1.03)	0.54		
smoking	1.04 (0.77–1.41)	0.81		
Hypertension	1.28 (0.88–1.87)	0.19		
Dyslipidemia	0.80 (0.59–1.08)	0.15		
Diabetes mellitus	1.79 (1.31–2.46)	< 0.01	1.62 (1.10–2.38)	0.01
Atrial fibrillation	0.83 (0.48–1.44)	0.50		
Prior myocardial infarction	1.09 (0.71–1.66)	0.69		
Prior heart failure	1.65 (1.16–2.36)	< 0.01	1.09 (0.71–1.71)	0.68
Clinical presentation				
ACS	1.02 (0.75–1.38)	0.91		
STEMI	1.04 (0.69–1.57)	0.84		
LVEF < 40%	2.67 (1.84–3.87)	< 0.01	1.80 (1.18–2.73)	< 0.01
Laboratory Data				
eGFR	1.00 (0.99–1.00)	0.55		
LDL cholesterol	0.80 (0.59–1.08)	0.15		
HbA1c	1.00 (0.89–1.13)	0.99		
CRP	1.06 (0.98–1.15)	0.13		
Prehospital medications				
ACE-i	1.96 (1.11–3.44)	0.02	1.44 (0.74–2.78)	0.28
ARB	1.28 (0.95–1.72)	0.11		
β blocker	1.11 (0.81–1.51)	0.52		
Calcium channel blockers	1.08 (0.80–1.46)	0.61		
Diuretics	1.36 (0.93–1.97)	0.11		
Statin	0.69 (0.50–0.94)	0.02	0.70 (0.48–1.01)	0.06
Warfarin	0.80 (0.44–1.44)	0.45		
DOAC	1.00 (0.14–7.20)	0.99		
Location of coronary artery lesions				
Left main coronary artery	1.24 (0.77–1.99)	0.38		
Left anterior descending artery	3.51 (2.03–6.06)	< 0.01	2.42 (1.28–4.58)	< 0.01
Procedural characteristics				
Multivessel disease	2.24 (1.62–3.10)	< 0.01	1.46 (0.98–2.18)	0.06
Calcification	2.21 (1.60–3.06)	< 0.01	2.26 (1.56–3.27)	< 0.01
CTO	1.09 (0.63–1.88)	0.77		
Long lesion (> 20 mm)	1.56 (1.15–2.12)	< 0.01	1.33 (0.93–1.92)	0.12
Small vessel (< 3.0 mm)	0.82 (0.60–1.13)	0.22		
Procedural success of PCI	0.67 (0.30–1.52)	0.34		

ACE-i = angiotensin converting enzyme inhibitor; ACS = acute coronary syndrome; ARB = angiotensin receptor blocker; CRP = C-reactive protein; DOAC = direct oral anticoagulants; eGFR = estimated glomerular filtration rate; HbA1C = hemoglobin A1C; LDL = low density lipoprotein; LVEF = left ventricular ejection fraction; STEMI = ST-elevation myocardial infarction CTO = chronic total occlusion; IABP = intra-aortic balloon pumping; PCI = percutaneous coronary intervention; STEMI = ST-elevation myocardial infarcti

To address potential bias from the high proportion of hemodialysis patients in our cohort, we conducted a sensitivity analysis excluding these individuals. The findings remained robust: the LAD group still exhibited significantly higher rates of MACE (p < 0.001), CV death (p = 0.011), TVR (p < 0.001), and all-cause mortality (p = 0.005). However, the difference in nonfatal MI (p = 0.122) between groups remained non-significant (Supplemental Fig. 1).

### Sensitivity analysis stratified by LAD single-vessel and LAD multivessel disease

We conducted a sensitivity analysis stratifying patients into Non-LAD, LAD (Only), and LAD (Multivessel) group**s **(Supplemental Fig. 2). Kaplan–Meier analysis showed significant differences in MACE, CV death, TVR, and all-cause death (all p < 0.001), but not in nonfatal MI (p = 0.100). For MACE and TVR, outcomes worsened progressively from Non-LAD to LAD (Only) to LAD (Multivessel). CV death and all-cause death were significantly higher in LAD (Multivessel) compared to both other groups, while Non-LAD and LAD (Only) did not differ significantly.

### ACS vs. Non-ACS

Considering the clinical presentation of ACS and CCS (Supplemental Fig. 3), the in-hospital mortality was significantly higher in patients in the LAD group with ACS (n = 387, 6.2%) than in those in (i) the LAD group with CCS (n = 599, 1.7%), (ii) non-LAD group with ACS (n = 89, 0%), and (iii) non-LAD group with CCS (n = 170, 0.6%) (LAD group with ACS vs. LAD group with CCS, p < 0.001; Supplemental Fig. 4). However, the presence or absence of ACS did not make a statistically significant difference in long-term all-cause death, MACE, TVR, or nonfatal MI in the LAD or non-LAD groups (Supplemental Fig. 4). In contrast, ACS only affected CV death in patients with LAD lesions (LAD group with ACS vs. LAD group with CCS, p = 0.042; non-LAD group with ACS vs. non-LAD group with CCS, p = 0.47; Supplemental Fig. 4).

### Impact of LMCA

To account for the potential confounding effect of LMCA lesions in the LAD group, this study compared short- and long-term outcomes between the LAD group with LMCA lesions, the LAD group without LMCA lesions, and the non-LAD group. The in-hospital mortality rate was higher in patients in the LAD group with LMCA lesions (7.1%) compared to those in the LAD group without LMCA lesions (3.0%) and the non-LAD group (0.4%) (LAD group with LMCA vs. LAD group without LMCA, p = 0.001). However, for the long-term prognosis, there were no significant differences in the rates of MACE, CV death, TVR, nonfatal MI, or all-cause death between patients in the LAD groups with and without LMCA lesions (Supplemental Fig. 5).

## Discussion

Previous studies have not reached a consensus on the prognostic advantages of PCI for LAD (especially proximal LAD lesions) and non-LAD lesions [6–8, 17, 18]. This study fills an important gap in the literature by specifically examining the prognostic impact of LAD lesions in patients who underwent their first PCI. The study found that patients with LAD lesions had worse outcomes than those without LAD lesions. This finding suggests that the presence of an LAD lesion may be a particularly important risk factor for adverse outcomes in patients undergoing PCI for the first time. This study highlights the need to carefully assess and manage LAD lesions in this patient population.

### Prognosis of patients with LAD lesions

Interestingly, the present study’s findings align with previous reports suggesting that the LAD is responsible for perfusing a significant proportion of the left ventricular myocardium [19–21]. This study also provides insight into the limited compensatory effect of collateral perfusion in RCA and LCx lesions compared to that of LAD lesions [4]. The study had certain limitations due to its retrospective nature, small sample size, and limited observation period. Nonetheless, the significant difference in prognosis between patients with and without LAD lesions indicates that patients with LAD lesions at the time of initial PCI are at a high risk of all-cause and CV death. It is important to note that the overall prognosis in the study was poorer than that reported in recent clinical trials in the era of drug-eluting stents [22]. This may be due to the inclusion of patients with more severe atherosclerosis, including those with LMCA lesions, STEMI, and regular hemodialysis, which have often been excluded from previous clinical trials. In addition, recent studies have shown that coronary plaques cluster proximally, especially in the LAD, correlating with worse outcomes [23–25]. This underscores the importance of considering plaque distribution when assessing risk and planning revascularization. Although our registry lacked detailed segmental data to subclassify LAD lesions, literature suggests that proximal segments often bear the greatest plaque burden and vulnerability, potentially explaining the poorer prognosis in our LAD group.

We conducted a supplementary analysis stratifying patients into Non-LAD, LAD (Only), and LAD (Multivessel) groups to assess the impact of LAD disease and overall atherosclerotic burden. This revealed a graded risk profile, with LAD (Multivessel) patients experiencing significantly worse outcomes compared to both Non-LAD and LAD (Only) groups. The stepwise increase in adverse events from Non-LAD to LAD (Only) to LAD (Multivessel) highlights the vulnerability of the LAD territory, especially in multivessel disease. These findings underscore the importance of LAD involvement and suggest an incremental risk associated with the extent of CAD.

### Prognostic impact of LMCA and ACS

In the present study, the patients with LAD lesions and ACS had a higher risk of in-hospital mortality than those without LAD lesions. Since ACS is a known prognostic factor [26], it is reasonable to assume that its presence negatively impacts the short-term prognosis of patients with LAD lesions. However, ACS did not affect long-term prognosis in the LAD or non-LAD groups. The better 1-year survival rate of patients with ACS in this study (7.1%) compared to previous reports (8.4–27.1%) may have influenced this result [27, 28]. In-hospital mortality was higher in the LAD group with LMCA lesions than in the LAD group without LMCA lesions. However, there was no significant difference in long-term prognosis between the two groups. This finding supports the notion that LAD lesions significantly impact the long-term prognosis after initial PCI, regardless of the presence of LMCA lesions.

### Interventions for LAD lesions

The introduction of drug-eluting stents has significantly reduced the need for repeat revascularization, making PCI the standard treatment for both CCS and ACS, including STEMI. However, the prognosis after PCI and CABG varies depending on the anatomical SYNTAX (Synergy between PCI with Taxus and Cardiac Surgery) score. It has been suggested that PCI has a short-term safety advantage, whereas CABG has a long-term advantage in terms of the risk of CV events [29, 30]. In the present study, LAD lesions were a significant determinant of clinical outcomes in patients with CAD compared to non-LAD lesions. This raises the question of whether PCI is appropriate for patients with significant stenosis or LAD occlusion. The outcomes of CABG performed at the same institution during the same period showed that patients who underwent CABG had comparable all-cause and CV mortality to those in the non-LAD group and better outcomes than those in the LAD group, regardless of the LMCA lesion (Supplemental Fig. 6). This suggests that a CABG revascularization approach may be appropriate for patients with CAD and LAD lesions. Additionally, patients with LAD lesions may benefit from a discussion regarding revascularization procedures by the cardiology team to improve their long-term prognosis.

### Limitations

The limitations of this study include its observational and single-center design, which may limit the generalizability of its findings. Potential bias related to antiplatelet therapy that could have influenced bleeding and ischemic events may have been present, as the type and duration of dual antiplatelet therapy were determined at the cardiologist's discretion. Myocardial viability was not assessed in patients with chronically occluded lesions, and those who underwent balloon angioplasty alone or with bare-metal stent implantation were not excluded. Follow-up coronary angiography was performed at the attending physician’s discretion, and less than half of the patients underwent follow-up angiography. The decision to perform TVR was also at the discretion of the treating physicians. Because the difference in MACE between the two groups was largely driven by TVR, this operator-dependent element should be considered when interpreting the results. This study evaluated the target vessel at the time of the initial PCI. However, it remains uncertain whether complete revascularization was achieved for any residual lesions in cases of multivessel disease. This is particularly relevant, as LMCA and LAD lesions have a high incidence of multivessel disease, and incomplete revascularization may impact long-term outcomes. Furthermore, because the specific reasons for deferring PCI were not systematically recorded, the impact of operator-driven decisions regarding revascularization completeness could not be fully assessed.

The SYNTAX score was not systematically calculated, limiting our analysis of CAD complexity between groups. Physiological assessments of coronary lesions were not uniformly performed, potentially introducing variability in stenosis severity evaluation. The high prevalence of hemodialysis patients may limit generalizability. This study was conducted between 2010 and 2015, which represents an earlier period of the DES era. Because this was a single-center, investigator-initiated study without external funding, long-term follow-up was not performed owing to limitations in personnel and financial resources.

## Conclusion

Among the patients with CAD, those with LAD lesions had more severe atherosclerotic profiles than those without lesions. LAD lesions were independently associated with poor long-term prognosis after PCI. Therefore, the cardiology team, which includes cardiologists and cardiac surgeons, may need to discuss the therapeutic strategies for revascularization in patients with LAD lesions.

## Supplementary Information

Below is the link to the electronic supplementary material.

Supplementary file1 (DOCX 4316 kb)

## Abbreviations

CAD: Coronary artery disease
LAD: Left anterior descending artery
MACE: Major adverse cardiovascular event
PCI: Percutaneous coronary intervention
TVR: Target vessel revascularization
