# Supplementary material for: Prognostic stratification of patients with coronary artery stenosis by presence or absence of left anterior descending artery lesions
Source: Cardiovasc Interv Ther. 2026 Mar 3;41(3):563–73. doi: 10.1007/s12928-026-01259-1 (PMC13279662; doi:10.1007/s12928-026-01259-1)
Supplement: Supplementary file 1 — Supplementary file1 (DOCX 4316 kb) [file 12928_2026_1259_MOESM1_ESM.docx]

**Supplemental Figures.**

**Supplemental Figure 1.**

**Clinical outcomes after excluding patients receiving hemodialysis. Kaplan–Meier curves compare the incidence of MACE, CV death, TVR, all-cause death, and nonfatal MI between patients in the LAD and non-LAD groups.**

CV, cardiovascular; LAD, left anterior descending artery; MACE, major adverse cardiovascular event; MI, myocardial infarction; PCI, percutaneous coronary intervention; TVR, target vessel revascularization.


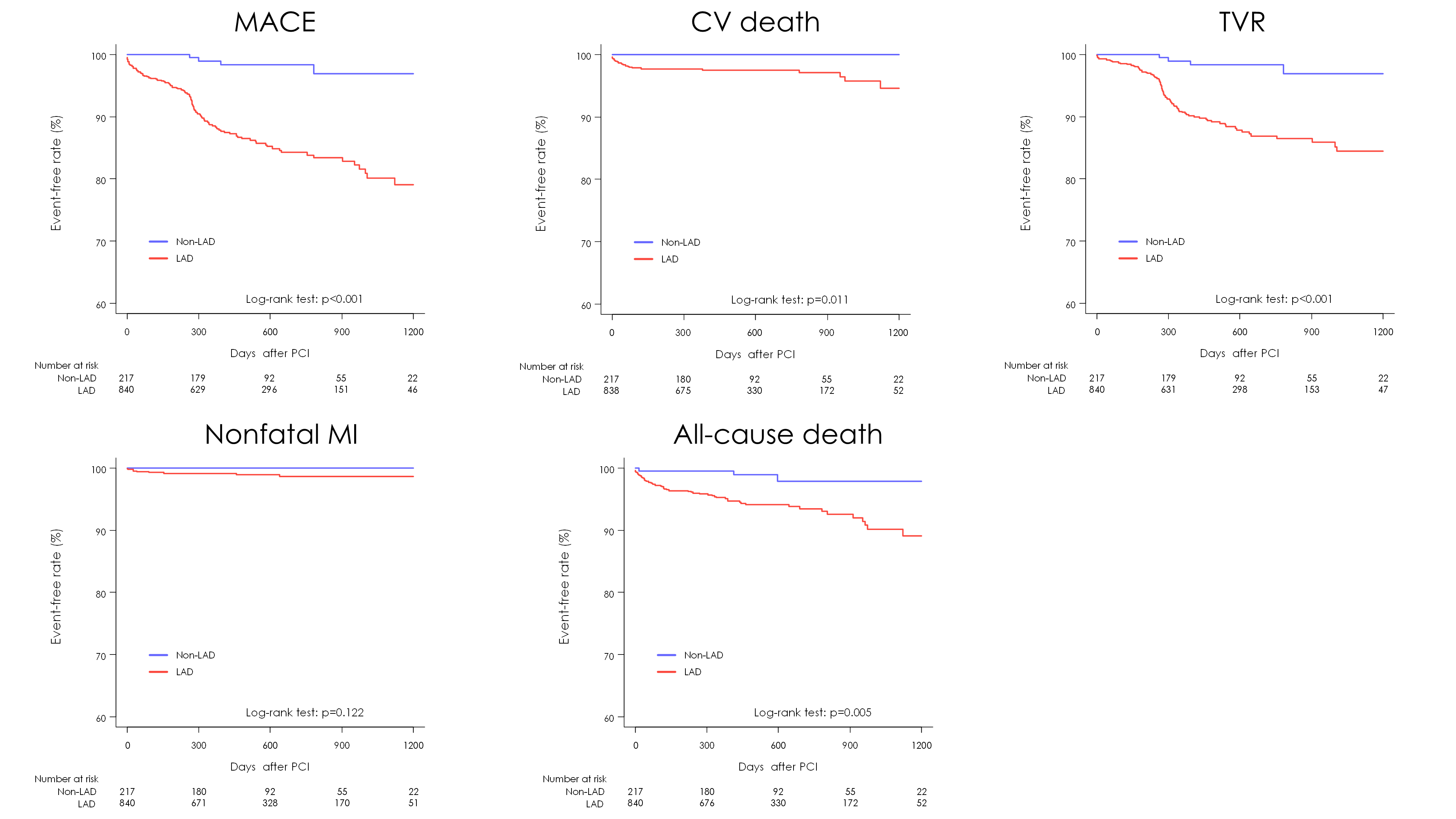


**Supplemental Figure 2.**

**Sensitivity analysis stratifying the cohort into three groups: Non-LAD, LAD(Only), and LAD(Multivessel). Kaplan–Meier curves demonstrate MACE, CV death, TVR, all-cause death, and nonfatal MI across the three groups.**CV, cardiovascular; LAD, left anterior descending artery; MACE, major adverse cardiovascular event; MI, myocardial infarction; PCI, percutaneous coronary intervention; TVR, target vessel revascularization.

**
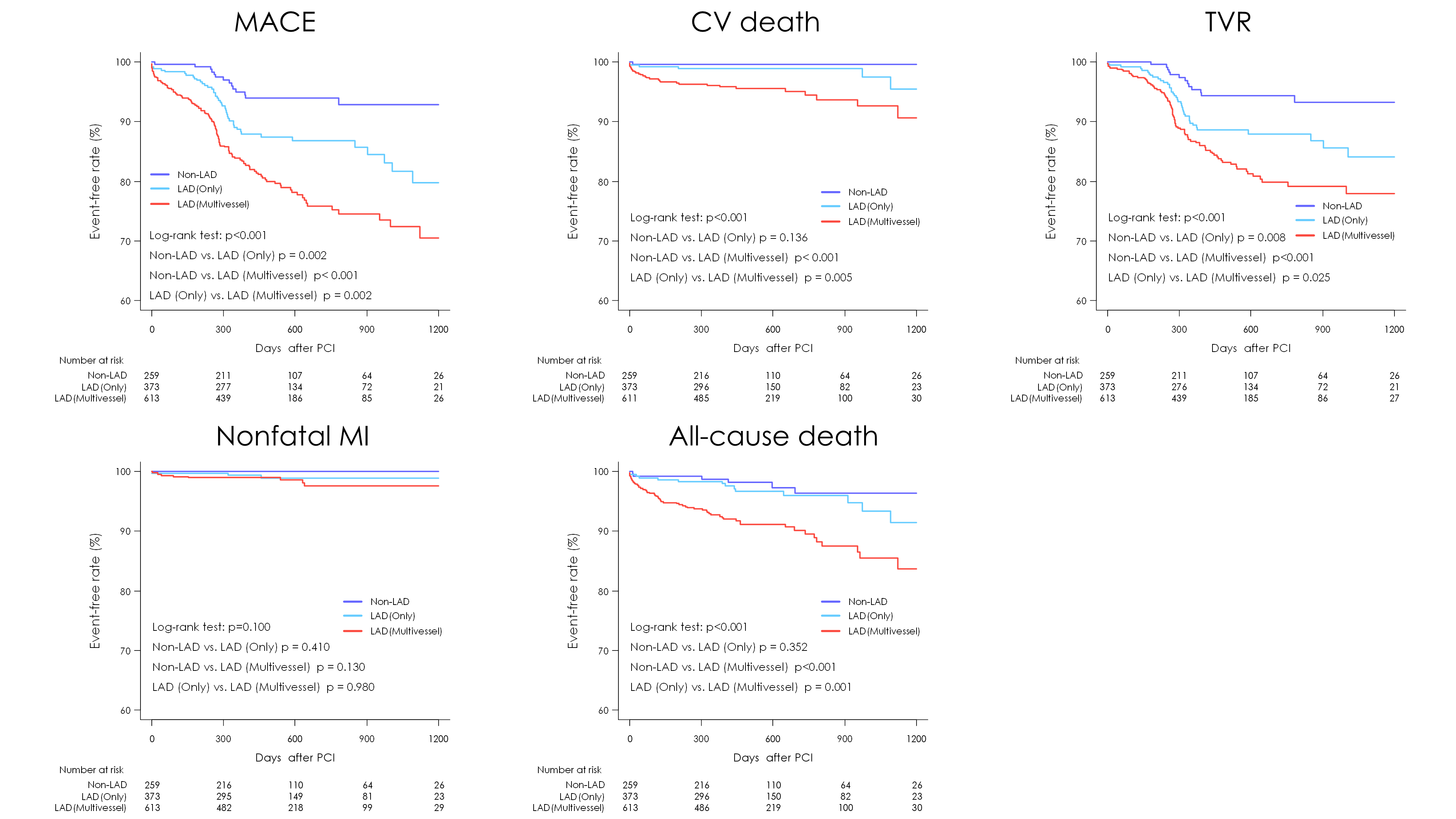
**

**Supplemental Figure 3.**

**Patient Flow and Stratification by LAD Lesion and Clinical Presentation.**
ACS, acute coronary syndrome; CABG, coronary artery bypass grafting; CCS, chronic coronary syndrome; LAD, left anterior descending artery; PCI, percutaneous coronary intervention


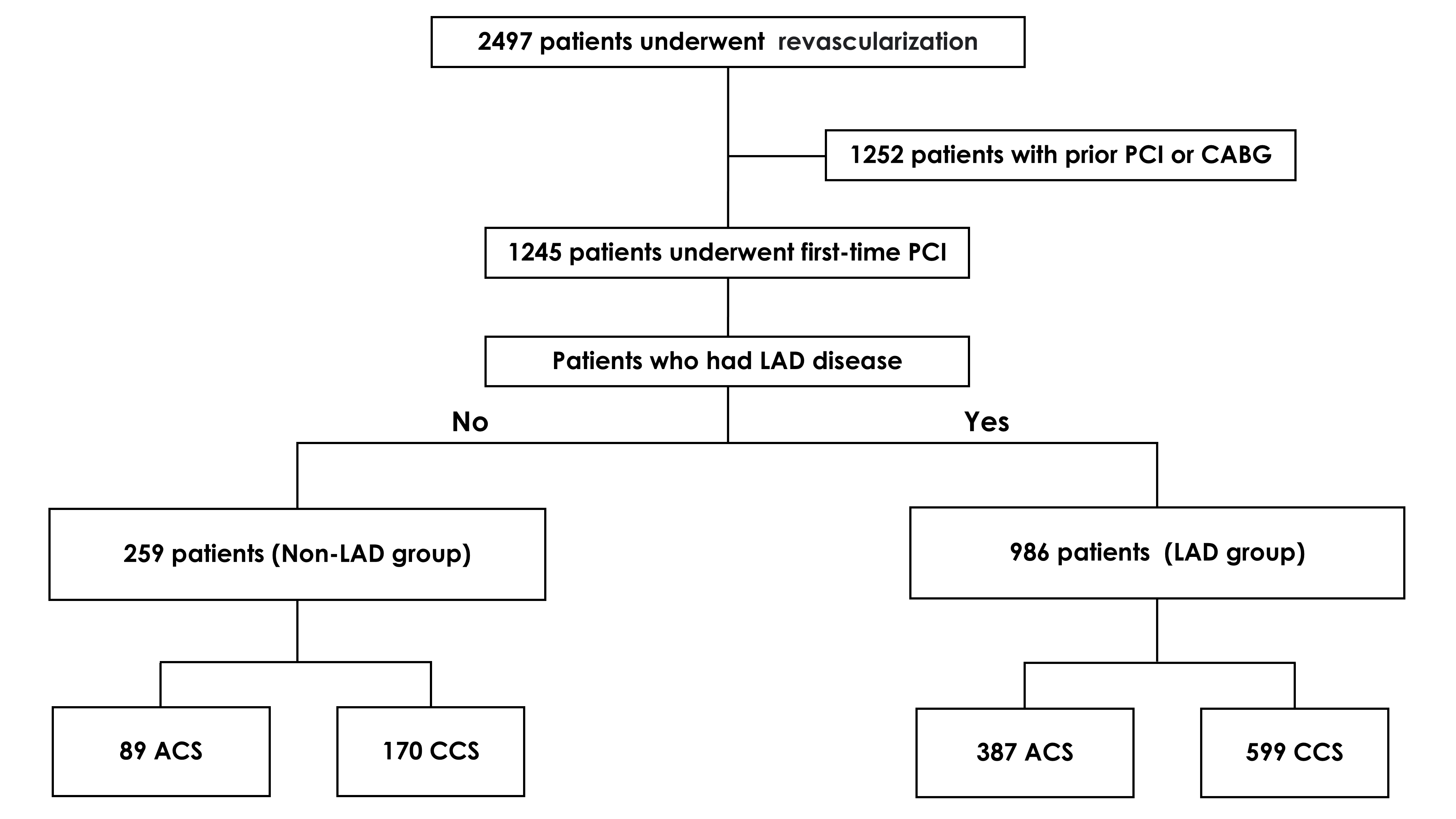


**Supplemental Figure 4.**

**Kaplan-Meier curves comparing all-cause death, MACE, TVR, and nonfatal MI between ACS and CCS patients within the LAD and non-LAD groups.**

ACS, acute coronary syndrome; CCS, chronic coronary syndrome; CV, cardiovascular; LAD, left anterior descending artery; MACE, major adverse cardiovascular event; MI, myocardial infarction; PCI, percutaneous coronary intervention; TVR, target vessel revascularization.

**
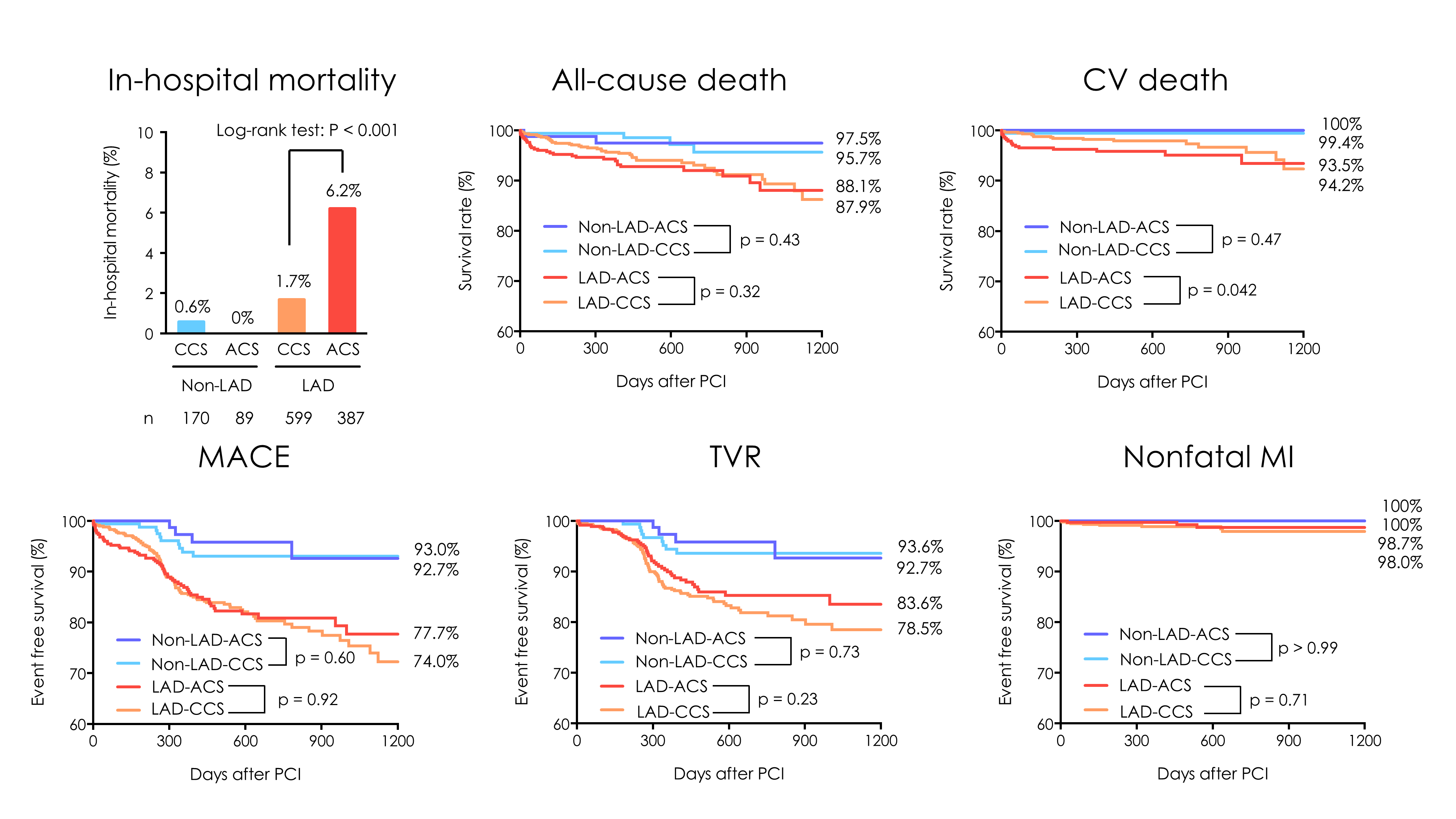
**

**Supplemental Figure 5.**

**Kaplan-Meier curves comparing short- and long-term outcomes among patients with LAD lesions involving LMCA, LAD lesions without LMCA involvement, and no LAD lesions.**CV, cardiovascular; LAD, left anterior descending artery; LMCA, left main coronary artery; MACE, major adverse cardiovascular event; MI, myocardial infarction; PCI, percutaneous coronary intervention; TVR, target vessel revascularization.

**
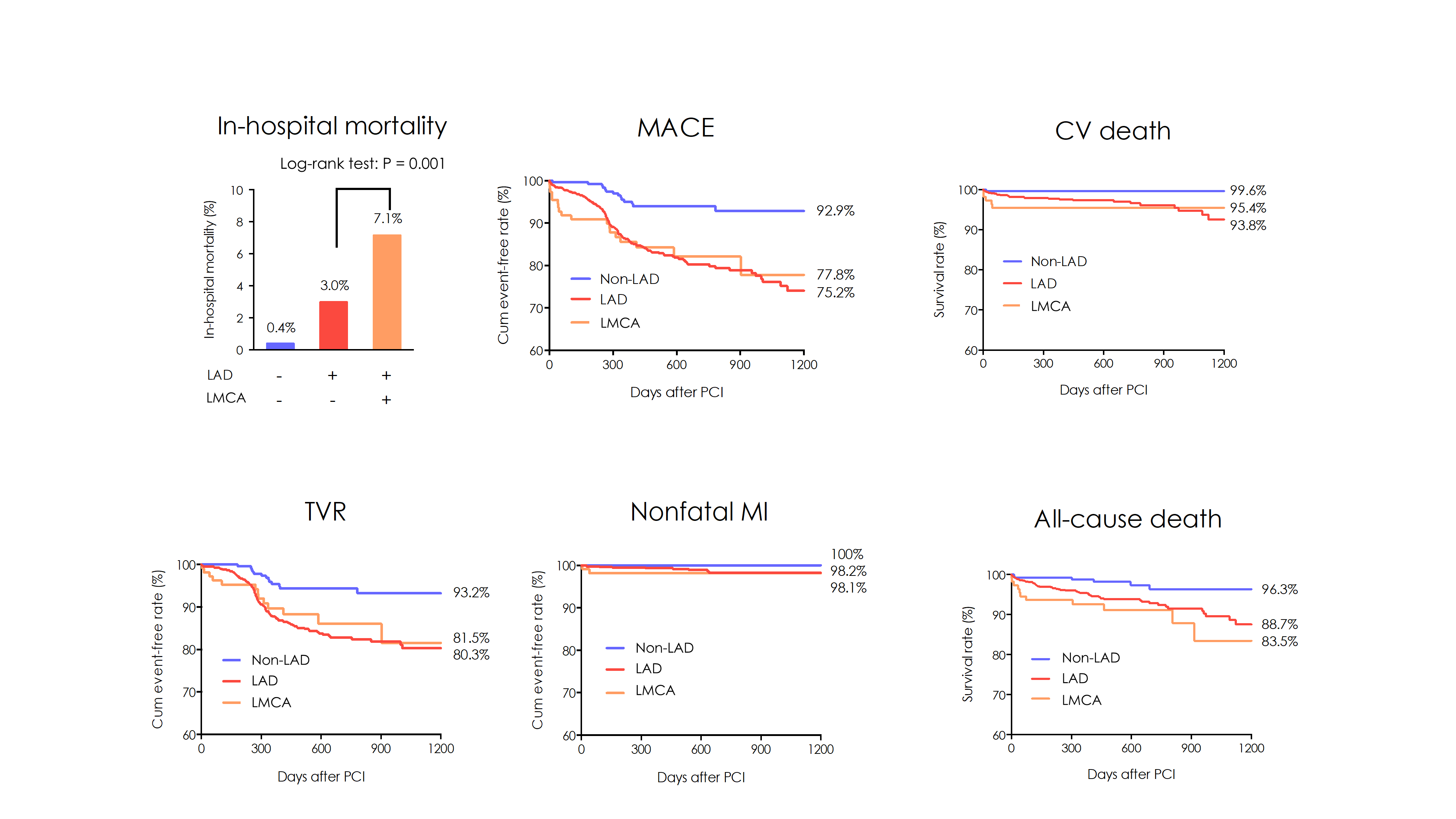
**

**Supplemental Figure 6.**

**Kaplan-Meier curves comparing outcomes among patients treated with CABG, PCI for non-LAD lesions, PCI for LAD lesions with LMCA involvement (LMCA+), and PCI for LAD lesions without LMCA involvement (LMCA-)**

CABG, coronary artery bypass grafting; CV, cardiovascular; LAD, left anterior descending artery; LMCA, left main coronary artery; MACE, major adverse cardiovascular event;

**
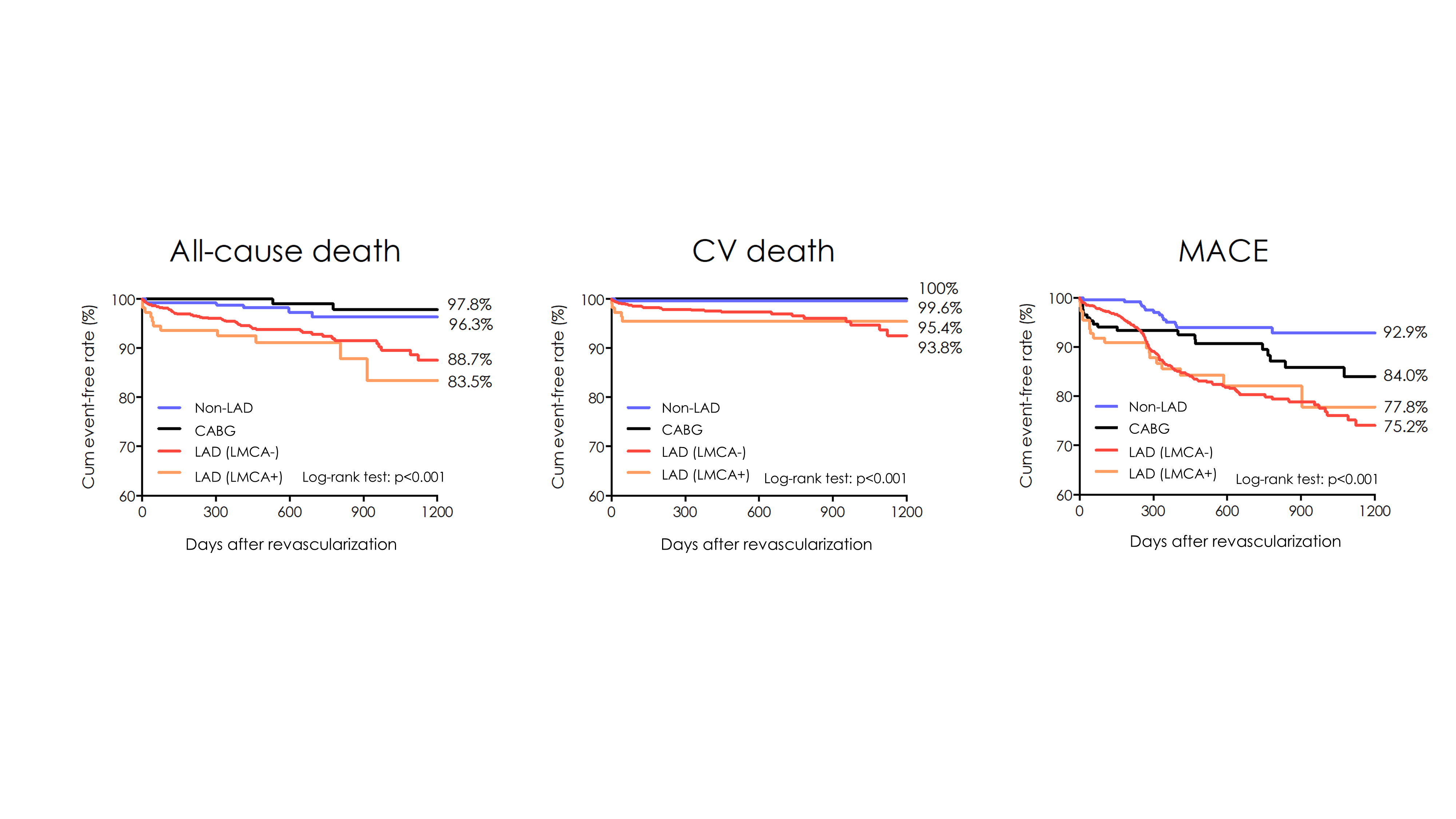
**
